# Supplementary material for: Serum Follicle-Stimulating Hormone Levels are Associated with Cardiometabolic Risk Factors in Post-Menopausal Korean Women
Source: J Clin Med. 2020 Apr 18;9(4):1161. doi: 10.3390/jcm9041161 (PMC7230188; doi:10.3390/jcm9041161)
Supplement: Supplementary file 1 [file jcm-09-01161-s001.pdf]

Supplementary Materials: Serum Follicle-Stimulating Hormone Levels Are Associated with Cardiometabolic Risk Factors in Post-Menopausal Korean Women

Eun-Soo Jung, Eun-Kyung Choi, Byung-Hyun Park and Soo-Wan Chae

Table S1. General characteristics of study participants.

Characteristics	Study population
N	609
Age (years)	53.51±2.94
Menopausal age (years)	50.25 ± 3.17
Alcohol (g/week)	18.95 ± 22.49
Blood pressure–lowering drugs (N, %)	21(3.45%)
KI (score)	34.21 ± 7.17
MRS (score)	31.83 ± 8.01
FSH (mIU/mL)	79.67 ± 24.36
Estradiol (pg/mL)	7.99 ± 9.38
Physical activity (METs/week)	2540.28 ± 3000.48
Height (cm)	157.37 ± 5.05
Weight (kg)	58.32 ± 7.35
BMI (kg/m^2)	23.54 ± 2.69
WC (cm)	83.32 ± 7.72
HC (cm)	93.14 ± 4.52
WHR	0.88 ± 0.06
SBP (mmHg)	119.43 ± 13.31
DBP (mmHg)	77.34 ± 10.21
Pulse (BPM)	70.98 ± 8.43
Glucose (mg/dl)	87.98 ± 7.91
Total cholesterol (mg/dl)	203.78 ± 32.40
Triglyceride (mg/dl)	115.65 ± 62.82
HDL-cholesterol (mg/dl)	56.68 ± 13.73
LDL-cholesterol (mg/dl)	124.57 ± 30.24
WBC (×10^3/μL)	4.95 ±1.18
RBC (×100^3/μL)	4.34 ± 0.31
Hemoglobin (g/dl)	13.29 ± 0.84
Hematocrit (%)	39.58 ± 2.35
Platelet (×10^3/μL)	243.42 ± 50.73
ALP (IU/l)	74.52 ± 17.02
γ-GTP (IU/l)	18.57 ± 12.34
AST (IU/l)	23.17 ± 5.39
ALT (IU/l)	21.47 ± 8.64
Total bilirubin (mg/dl)	0.82 ± 0.27
Total protein (g/dl)	7.52 ± 3.75
Albumin (g/dl)	4.45 ± 1.66
BUN (mg/dl)	14.67 ± 3.50
eGFR (ml/min/1.73 m^2)	112.03 ± 21.11
Uric acid (mg/dl)	4.68 ± 3.31
Total calcium (mg/dl)	9.52 ± 0.34
TSH (μIU/mL)	1.88 ± 0.96
hs-CRP (mg/l)	0.68 ± 2.23

Urine specific gravity	1.02 ± 0.01
Urine pH	6.13 ± 0.85

Values are presented as mean ± standard deviation (SD) for continuous variables or as a numerical proportion for categorical variables. KI, Kupperman index; MRS, menopause rating scale; FSH, follicle-stimulating hormone; BMI, body mass index; WC, waist circumference; HC, hip circumference; WHR, waist to hip ratio; SBP, systolic blood pressure; DBP, diastolic blood pressure; ALP, alkaline phosphatase; γ-GTP, γ-glutamyl transferase; AST, aspartate transferase; ALT, alanine transferase; BUN, blood urea nitrogen; eGFR, estimated glomerular filtration rate; TSH, thyroid-stimulating hormone; hs-CRP, high sensitivity C-reactive protein.

Table S2. Comparisons of dietary intake by quartiles of follicle-stimulating hormone.

	**Follicle-stimulating hormone (mIU/mL)**				***P* for trend 1)**	***P* 2)**
	**Q1 (≤62.20)**	**Q2 (62.21~76.87)**	**Q3 (76.88~94.00)**	**Q4 (≥94.01)**		
Calorie (kcal)	1559.51 ± 481.38	1490.42 ± 405.62	1490.41 ± 398.20	1474.53 ± 401.37	0.1007	0.3461
Carbohydrate (g)	245.58 ± 82.26	229.70 ± 65.96	237.67 ± 64.85	230.44 ± 64.16	0.2271	0.2068
Fat (g)	38.99 ± 19.21	38.20 ± 16.61	36.33 ± 17.57	37.71 ± 17.40	0.4743	0.6582
Vegetable fat	21.57 ± 12.99	19.57 ± 12.16	18.24 ± 10.25	20.15 ± 10.60	0.5605	0.1268
Animal fat	17.42 ± 11.10	18.62 ± 11.71	18.09 ± 12.66	17.56 ± 12.02	0.7672	0.7088
Protein (g)	60.94 ± 21.64	59.62 ± 20.42	58.88 ± 20.95	59.01 ± 20.14	0.4886	0.8456
Vegetable protein	31.82 ± 11.68	29.03 ± 10.35	30.31 ± 9.49	30.47 ± 8.94	0.9469	0.1633
Animal protein	29.12 ± 17.24	30.59 ± 16.46	28.57 ± 17.17	28.54 ± 16.49	0.7282	0.7088
Fiber (g)	20.97 ± 8.10	20.33 ± 6.50	21.38 ± 7.38	20.31 ± 7.55	0.7458	0.5590
Vitamin A (RE)	781.63 ± 460.29	800.05 ± 494.21	838.95 ± 415.49	804.12 ± 521.27	0.7322	0.7955
Vitamin D (μg)	4.31 ± 6.61	3.98 ± 5.59	3.91 ± 4.87	4.35 ± 6.15	0.8580	0.8895
Vitamin E (mg)	14.60 ± 7.27	14.01 ± 6.14	12.89 ± 5.79	13.36 ± 5.71	0.1365	0.1274
Vitamin K (μg)	204.49 ± 139.22	190.91 ± 106.81	215.75 ± 155.57	189.53 ± 159.10	0.2524	0.3638
Vitamin C (mg)	99.55 ± 50.75	99.51 ± 46.87	107.39 ± 58.56	99.58 ± 61.05	0.8347	0.5473
Thiamin (mg)	1.15 ± 0.42	1.12 ± 0.42	1.12 ± 0.47	1.13 ± 0.39	0.6651	0.9203
Riboflavin (mg)	1.03 ± 0.40	1.09 ± 0.69	1.05 ± 0.41	1.02 ± 0.40	0.7515	0.6039
Niacin (mg)	13.52 ± 5.30	13.87 ± 5.60	13.58 ± 5.27	13.74 ± 5.53	0.8801	0.9496
Vitamin B6 (mg)	1.44 ± 0.50	1.44 ± 0.50	1.48 ± 0.57	1.43 ± 0.53	0.8009	0.8689
Folate (μg)	451.05 ± 167.97	440.63 ± 162.62	462.96 ± 180.10	444.56 ± 167.36	0.8587	0.7086
Vitamin B12 (μg)	7.74 ± 6.76	8.09 ± 6.58	7.60 ± 5.27	8.12 ± 6.41	0.4921	0.8715
Pantothenic acid (mg)	4.16 ± 1.33	3.97 ± 1.38	4.08 ± 1.56	3.88 ± 1.44	0.1251	0.3806
Biotin (μg)	17.05 ± 9.67	15.85 ± 8.16	16.35 ± 8.96	15.96 ± 10.43	0.2052	0.7172
Calcium (mg)	460.51 ± 220.06	482.23 ± 222.87	487.43 ± 239.37	468.29 ± 225.60	0.8492	0.7528
Vegetable Ca	258.34 ± 115.63	247.68 ± 103.62	253.20 ± 110.65	247.68 ± 109.06	0.5078	0.8317
Animal Ca	202.17 ± 160.01	234.55 ± 176.00	234.23 ± 197.78	220.61 ± 169.81	0.5128	0.3975
Phosphorus (mg)	941.40 ± 306.11	950.11 ± 325.13	940.30 ± 304.84	937.73 ± 297.79	0.9831	0.9882
Sodium (mg)	3582.64 ± 1489.70	3704.80 ± 1528.29	3699.00 ± 1505.16	3538.33 ± 1401.90	0.9916	0.7221
Chlorine (mg)	313.22 ± 465.01	441.71 ± 536.41	427.15 ± 657.93	435.52 ± 637.91	0.4735	0.2284
Potassium (mg)	2712.64 ± 1043.14	2647.86 ± 831.39	2694.98 ± 940.68	2617.60 ± 1002.52	0.6113	0.8388
Magnesium (mg)	74.71 ± 44.84	79.18 ± 43.80	72.14 ± 49.44	72.85 ± 46.54	0.2872	0.5764
Fe (mg)	14.16 ± 6.31	14.37 ± 12.00	14.27 ± 4.69	13.24 ± 5.12	0.8413	0.5826
Vegetable Fe	10.72 ± 4.81	9.96 ± 6.02	10.84 ± 3.86	10.21 ± 4.15	0.5885	0.3630
Animal Fe	3.44 ± 2.89	4.42 ± 10.47	3.43 ± 2.22	3.03 ± 2.07	0.3505	0.2113

Zinc (mg)	9.01 ± 3.37	8.73 ± 2.94	9.15 ± 3.20	8.67 ± 2.66	0.8487	0.5064
Manganese (mg)	3.55 ± 1.24	3.39 ± 1.22	3.62 ± 1.30	3.43 ± 1.26	0.5930	0.4053
Iodine (μg)	486.25 ± 1183.87	375.10 ± 840.08	399.67 ± 914.26	444.91 ± 991.06	0.4263	0.7996
Selenium (μg)	82.50 ± 37.31	79.48 ± 35.59	74.86 ± 37.84	77.16 ± 35.15	0.2182	0.3612
Cholesterol (mg)	275.27 ± 181.81	281.31 ± 171.13	249.14 ± 146.32	264.07 ± 162.31	0.6584	0.3884
Total FA (g)	21.03 ± 11.91	23.55 ± 13.67	20.37 ± 10.77	21.23 ± 14.22	0.3942	0.4112
Saturated FA (g)	7.69 ± 5.03	8.12 ± 5.58	7.06 ± 4.16	8.07 ± 7.12	0.4799	0.6288
MUFA (g)	9.62 ± 6.52	9.72 ± 6.59	8.80 ± 5.30	10.24 ± 9.11	0.6518	0.6671
PUFA (g)	6.91 ± 4.07	6.19 ± 3.31	6.53 ± 3.69	6.55 ± 4.09	0.8549	0.7184
n-3 PUFA (g)	0.87 ± 1.47	0.66 ± 0.85	0.70 ± 0.86	0.90 ± 1.38	0.7413	0.4732

Values are presented as mean ± SD for continuous variables or as a numerical proportion for categorical variables. [1)] *P* for trend was calculated by analysis of variance (ANOVA, Jonckheere–Terpstra) tests. [2)] *P* was calculated by ANOVA. † Welch's ANOVA. MUFA, monounsaturated fatty acid; PUFA, polyunsaturated fatty acid.

Table S3. Associations between follicle-stimulating hormone and metabolic syndrome in postmenopausal women.

Metabolic syndrome	FSH	Unstandardized coefficients (B)	Odds ratio	*P*-value	95% CI	
					Lower	Upper
Model 1	Q1	0.987	2.682	0.014	1.226	5.868
	Q2	0.209	1.232	0.623	0.536	2.831
	Q3	0.564	1.757	0.181	0.769	4.015
	Q4		1.000			
Model 2	Q1	1.0320	2.807	0.011	1.268	6.212
	Q2	0.1968	1.217	0.646	0.526	2.819
	Q3	0.5513	1.736	0.195	0.755	3.992
	Q4		1.000			

Data were analyzed using logistic regression models with metabolic syndrome (Y/N) as the outcome and follicle-stimulating hormone as the explanatory variable. The results were expressed as odds ratios (95% confidence interval). The model 2 was adjusted for age and estradiol.

Table S4. Associations between follicle-stimulating hormone and components of metabolic syndrome in postmenopausal women.

		FSH	Unstandardized coefficients (B)	Odds Ratio	*P*-value	95% CI	
						Lower	Upper
Model 1	Hypertension	Q1	0.6320	1.881	0.019	1.109	3.191
		Q2	0.0863	1.090	0.763	0.623	1.909
		Q3	−0.0088	0.991	0.976	0.565	1.738
		Q4		1.000			
	Obesity	Q1	0.7957	2.216	0.001	1.361	3.608
		Q2	0.0348	1.035	0.895	0.617	1.736
		Q3	−0.4339	0.648	0.125	0.372	1.129
		Q4		1.000			
	WC (central obesity)	Q1	0.9877	2.685	0.006	1.321	5.459
		Q2	0.2453	1.278	0.459	0.668	2.445
		Q3	0.1092	1.115	0.748	0.573	2.172
		Q4		1.000			
	HDL-C	Q1	0.5480	1.730	0.044	1.015	2.947
		Q2	−0.2091	0.811	0.480	0.454	1.450
		Q3	0.1144	1.121	0.688	0.642	1.960
		Q4		1.000			
Model 2	Hypertension	Q1	0.5491	1.732	0.045	1.013	2.962

	Q2	0.0053	1.005	0.985	0.570	1.773
	Q3	–0.0411	0.960	0.887	0.545	1.691
	Q4		1.000			
Obesity	Q1	0.7173	2.049	0.005	1.249	3.361
	Q2	–0.0388	0.962	0.884	0.570	1.623
	Q3	–0.4745	0.622	0.096	0.356	1.088
	Q4		1.000			
WC (central obesity)	Q1	1.0971	2.995	0.004	1.428	6.284
	Q2	0.1786	1.195	0.595	0.619	2.309
	Q3	0.0377	1.038	0.913	0.528	2.042
	Q4		1.000			
HDL-C	Q1	0.5176	1.678	0.061	0.976	2.885
	Q2	–0.1886	0.828	0.527	0.462	1.485
	Q3	0.1330	1.142	0.642	0.652	2.000
	Q4		1.000			

Data were analyzed using logistic regression models with components of metabolic syndrome (Y/N) as the outcome and follicle-stimulating hormone as the explanatory variable. The results were expressed as odds ratios (95% confidence interval). Model 2 was adjusted for age and estradiol.
